# Causal pathways to social and occupational functioning in the first episode of schizophrenia: uncovering unmet treatment needs

**DOI:** 10.1017/S0033291721003780

**Published:** 2023-04

**Authors:** Kathleen Miley, Piper Meyer-Kalos, Sisi Ma, David J. Bond, Erich Kummerfeld, Sophia Vinogradov

**Affiliations:** 1 Department of Psychiatry and Behavioral Sciences, University of Minnesota, Minneapolis, MN, USA; 2 School of Nursing, University of Minnesota, Minneapolis, MN, USA; 3 Institute for Health Informatics, University of Minnesota, Minneapolis, MN, USA

**Keywords:** Early schizophrenia, motivation, social cognition, causal discovery, functional outcomes, machine learning

## Abstract

**Background:**

We aimed to identify unmet treatment needs for improving social and occupational functioning in early schizophrenia using a data-driven causal discovery analysis.

**Methods:**

Demographic, clinical, and psychosocial measures were obtained for 276 participants from the Recovery After an Initial Schizophrenia Episode Early Treatment Program (RAISE-ETP) trial at baseline and 6-months, along with measures of social and occupational functioning from the Quality of Life Scale. The Greedy Fast Causal Inference algorithm was used to learn a partial ancestral graph modeling causal relationships across baseline variables and 6-month functioning. Effect sizes were estimated using a structural equation model. Results were validated in an independent dataset (*N* = 187).

**Results:**

In the data-generated model, greater baseline socio-affective capacity was a cause of greater baseline motivation [Effect size (ES) = 0.77], and motivation was a cause of greater baseline social and occupational functioning (ES = 1.5 and 0.96, respectively), which in turn were causes of their own 6-month outcomes. Six-month motivation was also identified as a cause of occupational functioning (ES = 0.92). Cognitive impairment and duration of untreated psychosis were not direct causes of functioning at either timepoint. The graph for the validation dataset was less determinate, but otherwise supported the findings.

**Conclusions:**

In our data-generated model, baseline socio-affective capacity and motivation are the most direct causes of occupational and social functioning 6 months after entering treatment in early schizophrenia. These findings indicate that socio-affective abilities and motivation are specific high-impact treatment needs that must be addressed in order to promote optimal social and occupational recovery.

## Introduction

Schizophrenia and other psychotic disorders are characterized by severe disability in social and occupational functioning (Wiersma et al., 2000), reducing quality of life and causing significant societal burden (Cloutier et al., 2016). Though these impairments are evident early, specialized early intervention services have failed to drastically alter long-term functional outcomes (Norman et al., 2018; Secher et al., 2015). Indeed, the greatest improvements in social and occupational functioning (when present) are observed during the first 6 months of treatment, with little significant gain thereafter (Humensky, Essock, & Dixon, 2017; Phahladira et al., 2020). While numerous predictors of functional outcome have been identified [including positive and negative symptoms, cognitive impairment, and duration of untreated psychosis (DUP), to name a few] (Fett et al., 2011; Milev, Ho, Arndt, & Andreasen, 2005; Nakagami, Hoe, & Brekke, 2010; Penttilä, Jaä̈skel̈ainen, Hirvonen, Isohanni, & Miettunen, 2014; Santesteban-Echarri et al., 2017), some of them, such as positive symptoms, do not robustly affect functional outcomes even when they are altered by treatment (Swartz et al., 2007; Wunderink, Nieboer, Wiersma, Sytema, & Nienhuis, 2013). This suggests that many clinical predictors of functional outcomes *may not necessarily cause* functional outcomes.

Previous research identifying predictors of functional outcomes have been limited by statistical methods that are not informed by causal theory. Identifying treatment needs with the greatest potential impact requires identifying which predictors are *most plausibly causes* of functional outcomes. Causal modeling algorithms are novel methods which combine graph theory, statistics, and machine learning to produce hypothetical causal models (Spirtes, Glymour, & Scheines, 2000). Such models have been demonstrated to outperform predictive statistical approaches, such as structural equation models (SEMs) and other regression-based methods in the identification of treatment targets (Maathuis, Kalisch, & Bühlmann, 2009; Shen, Ma, Vemuri, & Simon, 2020; Stekhoven et al., 2012; Taruttis, Spang, & Engelmann, 2015). SEM analysis, for example, relies on *a priori* model selection to test a specific causal structure, and the effect estimate resulting from SEM is accurate only when the hypothesized causal structure is accurate, a condition hard to guarantee. SEM also requires specifying the causal structure of all included variables, which is rarely known in a complex causal system containing many variables. In contrast, the causal discovery analysis employed here uses a data-driven approach to search the space of possible SEMs to identify the causal network structure that best fits the data, including identification of causal effects of latent (unmeasured) variables (Ogarrio, Spirtes, & Ramsey, 2016). Thus, in addition to generating statistically defensible models of underlying causal structures (Shen et al., 2020), causal discovery offers the advantage of modeling a more comprehensive set of treatment target candidates.

In order to identify plausible causes of functional outcomes during the 6-month critical window for early intervention, we applied a novel data-driven causal modeling method to data from the Recovery After an Initial Schizophrenia Episode Early Treatment Program (RAISE-ETP) trial. Our aim was to identify variables that are modeled *to cause, rather than only predict or associate with*, social and occupational functioning, and thus represent high impact unmet treatment needs for promoting functional recovery in early schizophrenia. We validated our results in an independent dataset drawn from the Clinical Antipsychotic Trials of Intervention Effectiveness (CATIE) study.

## Methods

### Data acquisition

De-identified data were obtained from the National Institute of Mental Health National Database for Clinical Trials (https://data-archive.nimh.nih.gov/). The University of Minnesota Institutional Review Board approved this research as exempt from human subjects research.

### Participants

Participants for study 1 were drawn from the RAISE-ETP randomized controlled trial (Kane et al., 2016). RAISE-ETP enrolled 404 participants across 21 states. Inclusion criteria were: ages 15–40; diagnosis of schizophrenia, schizoaffective disorder, schizophreniform disorder, brief psychotic disorder, or psychosis not otherwise specified; only one lifetime episode of psychosis; and duration of antipsychotic use less than 6 months. RAISE-ETP participants who completed baseline and 6-month follow up assessments were included in this analysis (*N* = 276). Participants from both arms of the RAISE-ETP trial were included because we sought to identify variables that causally impact functional outcome regardless of treatment program; the treatment arm was included in the model to assess any effect on the estimated causal network structure.

Participants for study 2 were drawn from the CATIE trial (Stroup et al., 2003). CATIE enrolled 1600 participants across 50 sites; inclusion criteria were: ages 18–65 years and a schizophrenia diagnosis. Exclusion criteria were psychotic symptoms for less than 3 years and antipsychotic use less than 1 year. We studied CATIE participants who were 40 years of age and younger, had antipsychotic duration of less than 5 years, and who completed baseline and 6-month assessments of our primary outcome measures; our goal was to attain similarity with the RAISE-ETP participants while maximizing the sample size (*N* = 187).

### Study 1: Variables and measures

The outcome variables for our analysis included social and occupational functioning derived from the interviewer-rated Heinrichs–Carpenter Quality of Life Scale (QLS) (Heinrichs, Hanlon, & Carpenter, 1984), which was the primary outcome measure in the RAISE-ETP trial. Consistent with RAISE-ETP, we used the traditional subscale constructs for the Interpersonal Relations (items 1–8, assessing relationships and level of social activity) and Instrumental Role (items 9–12, assessing level of occupational role functioning, accomplishment, underemployment, and satisfaction with occupational functioning) subscales to derive our variables for Social and Occupational Functioning, respectively.

### Primary variables

Primary variables were selected based on previous findings that motivation, social cognition, cognition, negative symptoms and DUP are consistent predictors of functional outcome in schizophrenia (Fett et al., 2011; Milev et al., 2005; Nakagami et al., 2010; Penttilä et al., 2014; Robertson et al., 2014; Santesteban-Echarri et al., 2017).

*Motivation* was derived from three items from the Intrapsychic Foundations subscale of the QLS: item 13, ‘sense of purpose,’ item 14, ‘degree of motivation,’ and item 15, ‘curiosity.’ These items were originally derived as a measure of intrinsic motivation (Nakagami, Xie, Hoe, & Brekke, 2008) and may also reflect a general trait-like motivation (Choi, Choi, Felice Reddy, & Fiszdon, 2014). They have been used extensively as a measure of motivation in schizophrenia with demonstrated convergent validity with interviewer-rated measures of motivation and laboratory measures of effort-based decision making (Fervaha, Foussias, Takeuchi, Agid, & Remington, 2015; Horan et al., 2015). A recent factor analysis found that these items loaded most strongly on the Intrapsychic Foundations/Motivation subscale (Mueser et al., 2017), and they predict cross-sectional and longitudinal functional outcomes in early and chronic schizophrenia (Choi et al., 2014; Fervaha, Foussias, Agid, & Remington, 2015; Saperstein, Fiszdon, & Bell, 2011).

*Socio-affective capacity.* As there are no measures of social cognition in RAISE-ETP, we derived the variable ‘socio-affective capacity’ from the QLS as a proxy measure for higher order social cognitive abilities, such as social perception, theory of mind, and empathy. This measure consisted of item 20 ‘capacity for empathy’ and item 21, ‘capacity for engagement and emotional with the interviewer’ from the Intrapsychic Foundations subscale; they assess an individual's ability to perceive and respond to another person's perspective and affective state. Social perception, theory of mind, and empathy rely on partially shared neural circuitry and depend on intact functioning of the social brain (Green, Horan, & Lee, 2015; Shamay-Tsoory, 2011; Sparks, Mcdonald, Lino, O'donnell, & Green, 2010).

*Cognition.* A cognitive composite standardized *Z*-score was created from the Brief Assessment of Cognition using the average score of the six assessed cognitive domains (Keefe et al., 2004).

*Negative symptoms* were derived from the Positive and Negative Symptom Scale traditional three syndrome model composite score (Kay, Fiszbein, & Opler, 1987).

*DUP,* defined as time between onset of psychosis and initiation of antipsychotic medication, was measured in days at the time of study entry, and was log transformed due to skewness.

### Secondary variables

Our analysis included psychopathology, substance use, psychological well-being (Browne et al., 2016), attitudes toward medications, age (log transformed) and treatment arm (Table 1) as additional variables which could influence functional outcomes and to identify more determinate edges among the primary variables (Ogarrio et al., 2016). Variable descriptions are included in the Supplemental Methods. Like the primary RAISE-ETP study, we did not include prescribed chlorpromazine equivalents in this analysis.

**Table 1.** Study sample characteristics and variable descriptive statistics

	Study 1 (*N* = 276)		Study 2 (*N* = 187)	
	Mean (s.d.) or %		Mean (s.d.) or %	
**Demographics**				
Age (years)^a^	23.0	(4.9)	27.3	(6.1)
Gender: male	74.3		80.2	
Race				
American Indian or Alaskan Native	5.8			
Asian	2.9			
Black	35.1			
Native Hawaiian or Pacific Islander	0.4			
White	55.8		64.7	
Ethnicity: Hispanic or Latino	18.5			
**Baseline variables**				
Treatment assignment to coordinated specialty care	43.1			
QLS social functioning	19.9	(8.6)	21.4	(10.5)
QLS occupational functioning	5.7	(6.7)	6.7	(5.3)
QLS motivation	7.7	(3.6)	8.4	(4.1)
QLS socio-affective capacity	8.0	(2.2)	8.0	(2.6)
Neurocognitive composite *Z* score^b^	0.04	(1.0)	0.3	(0.9)
PANSS positive symptoms	18.9	(5.2)	18.0	(5.1)
PANSS negative symptoms	20.1	(5.2)	21.1	(6.7)
PANSS general symptoms	37.6	(7.7)	37.4	(8.8)
Calgary Depression Scale for schizophrenia	4.4	(3.9)	4.5	(4.0)
Clinical global impressions-severity	4.1	(0.8)	4.0	(1.0)
Duration of untreated psychosis (days)	185.7	(271.9)		
Days of alcohol use in last month	1.9	(4.7)		
Days of cannabis use in last month	3.2	(7.6)		
Well-being scale total score	4.0	(0.9)		
Mental health recovery measure total score	73.3	(18.1)		
Stigma Scale total score	4.0	(1.2)		
Brief evaluation of medication influences and beliefs total score	4.9	(1.0)		
Years of antipsychotic treatment			2.4	(1.6)
Face emotion discrimination task correct responses			24.9	(3.7)
**Six-month variables**				
QLS social functioning	22.9	(9.8)	23.7	(10.7)
QLS occupational functioning^a^	9.0	(7.5)	7.5	(5.8)
QLS motivation	8.6	(3.6)	8.8	(4.0)
QLS socio-affective capacity	8.0	(2.4)	8.2	(2.6)

a Age *p* = <0.001, *t* = −8.0; QLS occupational functioning *p* = <0.001, *t* = 4.0.

b Neurocognitive tests utilized differed between groups and means were not compared. Study 1 utilized the Brief Assessment of Cognition and study 2 utilized a customized panel of neurocognitive tests. PANSS = Positive and Negative Symptom Scale, QLS = Quality of Life Scale; Blank cells indicate variable was not available in dataset.<

### Study 2 variables and measures

Study 2 used the same variables as study 1 for social functioning, occupational functioning, motivation, and socio-affective capacity and several of the same secondary variables, as detailed in Table 1.

### Statistical analysis

Independent sample *t* tests for continuous variables and Pearson's chi-square tests for categorical variables tested for differences in demographic and clinical variables between (1) RAISE-ETP participants included and excluded from study 1, and (2) participants in study 1 and study 2.

**Fig. 1.**
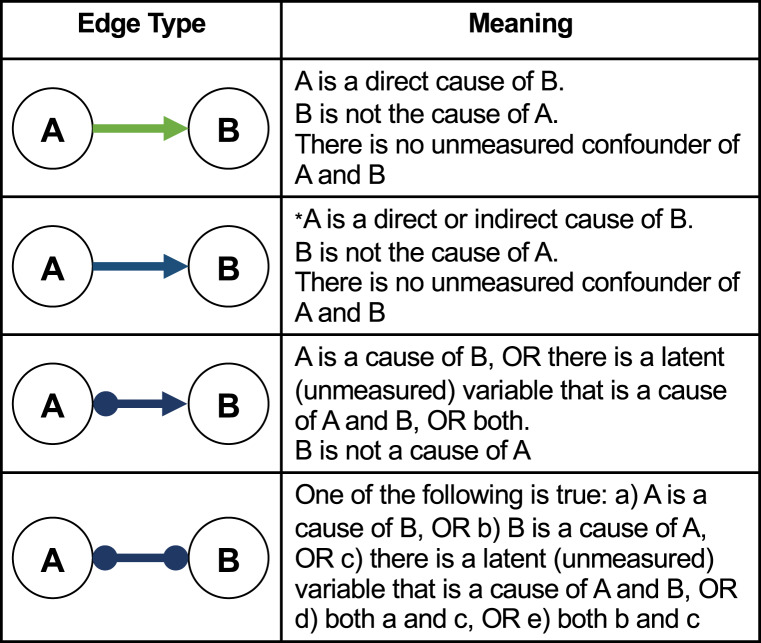
Greedy fast causal inference edge types and meaning. *Note: When viewed in Tetrad 6.6.0, this edge type is represented as a thin green edge. We have used a blue edge here to distinguish the edge more easily from the thick green directed edge above.

We used the Greedy Fast Causal Inference (GFCI) algorithm to estimate the causal relationships among baseline and 6-month variables. GFCI searches the space of all possible partial ancestral graphs (PAGs) using a combination of goodness of fit statistics and conditional independence tests to identify the PAG that best models the causal process from which the data were sampled (Chickering, 2002; Ogarrio et al., 2016; Ramsey, 2015). In its first step, GFCI temporarily assumes that there are no unmeasured common causes of the observed variables and searches the space of all possible causal models to find the model which has the best penalized likelihood score. In its second step, it drops the assumption that there are no unmeasured common causes, and refines the graph produced by the first step by testing for all possible statistical inconsistencies that could have been induced by latent common causes. These inconsistencies are identified using conditional independence tests, and the graph is modified appropriately (Chickering, 2002; Ogarrio et al., 2016; Ramsey, 2015). In the resulting PAG, variables are represented as nodes in the graph. The type and orientation of an edge connecting two nodes specifies the nature of the modeled causal relationship (Fig. 1). See the Supplementary Methods and citations (Chickering, 2002; Ogarrio et al., 2016; Ramsey, 2015) for more detailed information on GFCI, including assumptions and proof of correctness. This process has been mathematically proven to be asymptotically correct, and has outperformed similar methods in simulations, where adjacencies and edge orientations in the output PAG have been shown to have high precision and recall on sample sizes comparable to the datasets studied here (Ogarrio et al., 2016).

The Tetrad software package version 6.6 was used for the GFCI analysis (https://cmu-phil.github.io/tetrad/manual/). Background knowledge included that the 6-month variables could not cause baseline variables, and no variable could cause age. Model parameters include a Bayesian Information Criterion score, set to the standard penalty discount value of 1, and Fisher *Z* test, set to the default value of 0.01.

### Effect size estimation and model fit statistics

Raw and standardized effect sizes (ES) of the model-identified causal relationships were estimated by fitting a linear SEM to the PAG. Edges connecting to a possible latent variable were modeled as indirect covariations for the purposes of the SEM. The Comparative Fit Index (CFI) and Root Mean Square Error of Approximation (RMSEA) were inspected to assess model fit. The R package Lavaan 0.6.3 was used for this analysis (Rosseel, 2012). Due to the prevalence of multiple possible latent variables in the CATIE validation study, model parameters were not estimated to avoid producing a SEM with low interpretability.

### Graph stability

The stability of the study 1 and study 2 PAGs were evaluated by running GFCI on 1000 jackknifed datasets containing 90% of the original dataset and 1000 bootstrapped datasets. The percentage of edges in the PAG that were confirmed in re-sampled graphs was calculated. Additional SEM comparison and sensitivity analyses were performed to assess for effects of measurement or construct overlap (see Supplemental Methods).

## Results

### Participants

After excluding 128 participants for missing data, study 1 included 276 participants from RAISE-ETP (Table 1). Excluded participants had higher scores on the Beliefs About Medications scale (*t* = −2.16, *p* = 0.03). There were no statistically significant differences on any other baseline variables between participants included and excluded from the analysis (online Supplementary Table S1).

Study 2 included 187 participants from CATIE (Table 1). Descriptive statistics were not compared between included and excluded CATIE participants because the included subset was selected to best match the RAISE-ETP cohort. Study 1 participants were significantly younger than study 2 participants (*t* = −8.0, *p* < 0.001) and had better 6-month occupational functioning (*t* = 4.0, *p* < 0.001) (Table 1).

### Study 1: Primary analysis

**Fig. 2.**
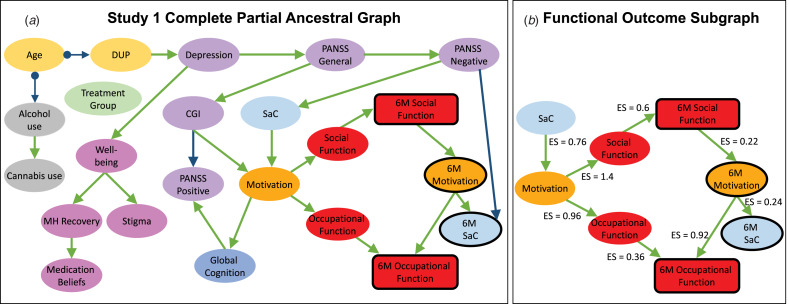
Study 1 complete PAG and functional outcome subgraph. All variables represent baseline unless specified as 6M (6 month). Alcohol use = days of alcohol use in previous month; Cannabis use = days of cannabis use in the past month; CGI = Clinical Global Impressions-Severity score; Depression = Calgary Depression Scale for Schizophrenia; DUP = duration of untreated psychosis (log transformed); Global Cognition = Brief Assessment of Cognition composite Z score; Medication Beliefs = Brief evaluation of medication influences and beliefs total score; MH Recovery = Mental Health Recovery Measure total score; PANSS = Positive and Negative Symptom Scale; SaC = Socio-affective capacity; Stigma = Stigma scale total score; Treatment Group = randomized into coordinated specialty care *v.* Treatment as Usual. Wellbeing = Well-being scale total score.

**Fig. 3.**
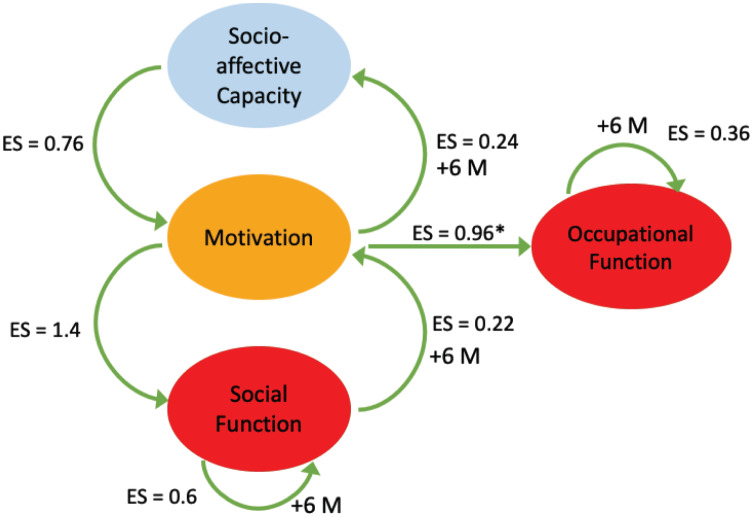
Study 1 augmented rolled graph. *Causal effect size for baseline motivation on baseline occupational functioning. The causal effect size from 6-month motivation to 6-month occupational functioning is 0.92. ES = effect size. + 6 indicates a 6-month time cycle over which the effect occurred.

The full PAG for the RAISE-ETP cohort is shown in Fig. 2*a*, and a functional outcome subgraph is shown in Fig. 2*b*. The strength and sign of modeled causal relationships in the functional outcome subgraph are provided in Fig. 3 and all effect sizes (raw and standardized) are provided in online Supplementary Table S2. The raw effect sizes are reported here. Fit statistics indicated an appropriate fit of the model to the data (CFI = 0.884; RMSEA = 0.066).

#### Causal pathways to 6-month social functioning

Our model contains one causal pathway leading to 6-month social functioning: baseline socio-affective capacity is a cause of motivation (ES = 0.77), which is then a cause of baseline social functioning (ES = 1.5), which is then a cause of 6-month social functioning (ES = 0.6).

#### Causal pathways to 6-month occupational functioning

Our model contains two causal pathways leading to 6-month occupational functioning. Path 1: baseline motivation is a cause of baseline occupational functioning (ES = 0.96), which then is a cause of 6-month occupational functioning (ES = 0.36). Path 2: 6-month social functioning is a cause of motivation (ES = 0.21), which in turn is a cause of occupational functioning (ES = 0.92).

#### Causal effects of cognition, negative symptoms, and DUP

Cognition is not included in a causal pathway to social or occupational functioning. Negative symptoms are a direct cause of socio-affective capacity at baseline (ES = −0.27), but their effect on social and occupational functioning is mediated by socio-affective capacity and motivation. DUP is upstream of the causal pathway to functional outcomes, and its effect is mediated by several variables including socio-affective capacity and motivation.

#### Six-month causal cycles

Our model contains several causal cycles which unfold over a period of 6 months, as shown in the augmented rolled graph (Fig. 3). For example, socio-affective capacity causes motivation at baseline (ES = 0.76), and at 6 months, motivation causes socio-affective capacity (ES = 0.24). Similarly, motivation causes social functioning at baseline (ES = 1.4), and at 6 months, social functioning causes motivation (ES = 0.22). Thus, those with better socio-affective capacity at baseline have better motivation, and sustained improvements in motivation at 6 months lead to improved socio-affective capacity: a virtuous cycle. Motivation and social functioning also form a virtuous cycle: those with better motivation at baseline consequently enjoy better social functioning, which feeds forward to better motivation at 6 months.

### Study 1: Graph stability

The jackknife had 100% concordance with all graph features (i.e. edge presence, edge absence and edge orientation) in the functional outcome subgraph and the full PAG. The bootstrap had 100% concordance with all features in the functional outcomes subgraph (online Supplementary Table S3). We also tested a model in which the motivation and socio-affective capacity variables covary instead of being separate variables with the causal relationships detailed above, however our original model had better fit (*p* < 0.0001, see Supplemental Methods). This is expected because GFCI is capable of modeling unmeasured common causes, therefore if motivation and socio-affective capacity are better represented by a single factor GFCI should indicate the possibility of a latent variable.

### Study 2: Primary analyses

**Fig. 4.**
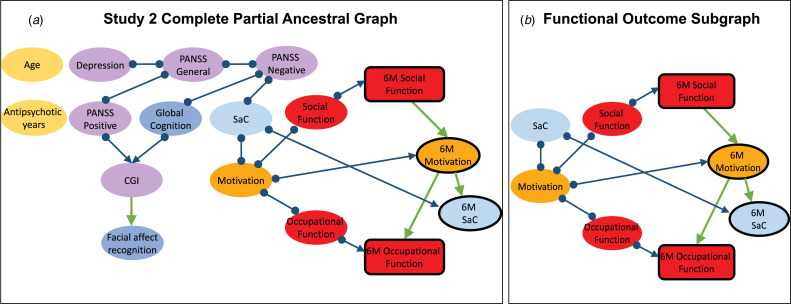
Study 2: validation complete PAG and functional outcome subgraph. All variables represent baseline unless specified as 6M (6-month). Antipsychotic years = years of antipsychotic use; CGI = Clinical Global Impressions-Severity score; Depression = Calgary Depression Scale for Schizophrenia; Facial Affect Recognition = Face emotion discrimination task correct responses; Global Cognition = Cognition composite Z score; PANSS = Positive and Negative Symptom Scale, SaC = Socio-affective capacity.

The overall structure of the PAG for the functional outcome subgraph in study 2 is similar to study 1 (Fig. 4). Relationships between baseline and 6-month socio-affective capacity, motivation, and social and occupational functioning variables are maintained in the study 2 PAG, supporting the findings from study 1. Some edge orientations in this graph are less determinate, which permits but does not confirm the corresponding orientations found in study 1.

### Study 2: Graph stability

The jackknife graph had 100% concordance with all graph features for the functional outcome subgraph. The bootstrap graph resulted in two dropped edges (connecting baseline to 6-month socio-affective capacity and baseline motivation to 6-month socio-affective capacity), and had more determinate edge orientations for two edges (connecting baseline to 6-month occupational functioning and baseline to 6-month social functioning) when compared to the functional outcome subgraph in the primary PAG (online Supplementary Table S3).

## Discussion

### Summary of causal pathway findings

We used causal discovery modeling on a large sample of patients with first episode schizophrenia, to model which variables *plausibly cause* social and occupational functioning 6 months after entering treatment. We discovered a primary causal pathway in our model with large effect sizes from baseline socio-affective capacity to motivation, and from motivation to both social and occupational functioning at 6 months. Interestingly, cognition did not play a causal role; and DUP and negative symptoms indirectly influenced social and occupational functioning. These results may indicate the importance of vigorously and specifically targeting *socio-affective capacity and motivation* as soon as possible after a young individual enters care. Further, the discovered causal 6-month cycle between socio-affective capacity and motivation suggests that early gains in these areas will be self-sustaining through a positive feedback loop. To the best of our knowledge, most evidence-based treatment programs do not explicitly identify these as critical targets of intervention nor directly assess them as part of patient outcome and program evaluation.

We also uncovered *a modeled causal cycle between social functioning and motivation*. At baseline, motivation was a cause of social functioning, and at 6 months social functioning was a cause of motivation. Higher motivation then led to higher occupational functioning. A recent SEM study using RAISE data found that social functioning, but not occupational functioning, predicted later motivation (Fulford et al., 2017). Directly improving *social functioning* may be a third critical early treatment target for maintaining motivation and ultimately improving occupational functioning (Fulford et al., 2017).

### Possible mechanisms for the causal pathways and cycles

#### Socio-affective capacity and motivation

Socio-affective capacity as measured in this study may capture aspects of social perception, theory of mind, and empathy during the QLS interview. As such, this measure may reflect social cognition abilities and the capacity to engage interpersonally. Shared neural mechanisms underlie social cognition and intact reward processing (necessary for adaptive motivated behavior), including important hubs in the ventromedial prefrontal cortex and the anterior cingulate (Fareri & Delgado, 2014); moreover, social stimuli are primary reinforcers for reward networks and activate frontal−striatal circuits (Fareri & Delgado, 2014). Deficits in perceiving and processing social stimuli and aberrant functioning of medial prefrontal circuitry can likely affect aspects of reward processing and motivated behavior. Consistently, we have found that social cognition training drives both adaptive changes in motivated behavior (Fisher et al., 2017; Miley et al., 2020) and medial prefrontal cortex neural activity (Subramaniam et al., 2012).

#### Motivation and social functioning

Individuals with psychotic disorders exhibit not only impaired social cognition (Fett et al., 2011), but reductions in social drive (Cornblatt et al., 2012; Tarbox & Pogue-Geile, 2008), and specific deficits in the valuation of social reward (Catalano, Heerey, & Gold, 2018). Poor motivation for social rewards may therefore drive impairments in social functioning. At the same time, social inclusion and adaptive social functioning are critical developmental accomplishments during the adolescent and young adult years (Blakemore, 2008; Filia, Jackson, Cotton, & Killackey, 2019; Filia, Jackson, Cotton, Gardner, & Killackey, 2018; Gardner, Filia, Killackey, & Cotton, 2019), coinciding with the typical age of onset of psychosis. Social exclusion during this period may lead to the development of socially defeatist beliefs, further impeding motivated behavior for social participation (Campellone, Sanchez, & Kring, 2016). Thus, directly targeting social functioning may lead to enriched social environments that reinforce social rewards and improve the valuation of these rewards from continued exposure to positive social experiences, resulting in improved motivated behavior. Improved motivation may in turn promote engagement in goal setting and pursuit of occupational endeavors, and feedback to social cognitive functioning via the modulation of shared neural networks which underlie social cognitive and reward processes.

### Causal roles of negative symptoms, cognitive impairment, and DUP

The negative symptoms of schizophrenia are strongly associated with poor functional outcomes (Milev et al., 2005; Robertson et al., 2014). While negative symptoms were in the causal pathway to functional outcome in our model, their effect was at least partially mediated by socio-affective capacity and motivation. Socio-affective capacity and motivation share some, but not full, conceptual overlap with negative symptoms as measured by the PANSS subscale, which also includes measures of blunted affect and alogia. That the effect of negative symptoms in our model was at least partially mediated by socio-affective processing and motivation may suggest that specifically targeting the *asociality* and *avolition* aspects of negative symptoms – which rely on intact socio-affective and motivation processes − may be most likely to drive improvements in both social and occupational functioning.

Unexpectedly, baseline cognitive impairment was not found to be in the causal pathway to functional outcomes, despite the large body of evidence linking cognition to functional outcomes (Fett et al., 2011; Santesteban-Echarri et al., 2017). It is possible that significantly impaired cognition causes poor functional outcomes, but that this relationship does not hold with only mildly or moderately impaired cognition, leading to unstable or missed relationships in causal discovery analyses. It is also possible that the relationship between cognition and functional outcomes is mediated by variables not measured in this study, such as more specific measures of social cognition, reward processing, or other neural/neurocognitive operations (Bhagyavathi et al., 2015; Gard, Fisher, Garrett, Genevsky, & Vinogradov, 2009; Green, Hellemann, Horan, Lee, & Wynn, 2012). In our validation study drawn from the CATIE trial, cognition was possibly on a causal pathway to functional outcomes, however the direction of these relationships cannot be determined. RAISE-ETP used a less comprehensive cognitive assessment battery compared to CATIE, and it is possible that this resulted in the observed differences between the two datasets.

The association of DUP with functional outcomes in schizophrenia has also been strongly supported by previous research (Penttilä et al., 2014); however, DUP was upstream in the causal pathways to functional outcomes in our model. It remains largely unknown whether shortening DUP will lead to improved long-term outcomes (Penttilä et al., 2014), and the specific mechanisms through which DUP impacts functional outcomes is unknown. Our model suggests that shortening DUP could impact functional recovery via its direct and downstream effects on several other clinical features, including depression, global disease severity, negative symptoms, motivation, and socio-affective capacity.

### Unmet treatment needs in early schizophrenia

Our results point to critical unmet therapeutic needs in current coordinated specialty care (CSC) early intervention services, which are now the gold standard treatment for first episode schizophrenia. While CSC programs indirectly aim to improve social functioning, they currently lack explicit and consistently delivered interventions which robustly target social cognition or motivation as primary outcomes. Yet, deficits in social cognition and motivation are malleable to targeted interventions in early schizophrenia, such as social cognitive training, social skills groups, recovery focused goal-setting and digitally delivered motivational coaching (Eack et al., 2009; Fernandez-Gonzalo et al., 2015; Fisher et al., 2017; Fulford, Piper Meyer-Kalos, & Kim Mueser, 2020; Roberts et al., 2014; Schlosser et al., 2018). Our results highlight the need for research on the integration of existing and novel social cognition and motivation interventions into CSC programs. Further, they suggest that specifically targeting social cognitive processes when individuals first enter treatment may exploit a positive feedback relationship between social cognition and motivation, thereby driving improved social and occupational functioning. Interventions specifically targeting social recovery have been shown to elicit improvements in social engagement when added to early intervention services (Fowler et al., 2018) and should continue to be evaluated as critical treatment components.

### Validation and limitations

Our validation study from the CATIE dataset did not contradict the results of our primary analysis and supported many of the findings. The overall structure of the functional outcome subgraph was very similar to the RAISE-ETP model even though many edge orientations in the graph were less definitive. The less definitive relationships could be due to differences in sample size, which can influence the ability of the GFCI algorithm to rule out alternative causal models. RAISE-ETP participants were younger and included individuals aged 15–17 who may have different supports and functional expectations than adult patients. CATIE participants had greater illness chronicity, longer duration of exposure to medications, and an established diagnosis of schizophrenia (*v.* one lifetime episode of psychosis), which could lead to more heterogeneity in symptoms and functioning. Temporally, enrollment dates for RAISE-ETP and CATIE were approximately a decade apart during a movement toward recovery-oriented care. It is likely that these differences could also impact results pertinent to longitudinal functional outcomes, including the directionality and/or strength of causal relationships among our primary variables. These differences may lend support to the value of our validation study, suggesting that our results could generalize beyond illness phase and contextual factors and are not simply attributed to specific features of the population studied.

Our study has several limitations. First, because RAISE-ETP did not include laboratory measures of social cognition, we relied on interviewer-rated proxy measures of socio-affective capacity and engagement with the interviewer, the construct validity of which is unknown. Until validated by future research, our interpretation of the relationship between socio-affective capacity, motivation and functional outcomes should be considered in the context of this limitation. While our motivation measure has been validated in previous research, it is similarly an interviewer-rated item. Future prospective research must build on our analyses and include well-validated and robust social cognition and motivation measures to confirm the causal influence of these domains on functional outcomes. We focused our analysis on clinical predictors (i.e. symptoms, cognition) that could be treatment targets, and did not include variables reflecting treatment engagement or prescribed antipsychotic dose equivalents, which could also impact short-term functional outcomes. Some variables had non-Gaussian distributions which could affect model performance. The primary method of analysis, GFCI, was recently developed, and although some benchmarking has been done, its finite-sample performance has not been completely characterized. GFCI generates a model of the plausible causal relationships from which the data were sampled, and its correctness relies on some assumptions that cannot be directly tested, but are plausible for the studied data. Statistical models can be wrong, however they are highly useful if accurate. GFCI also cannot identify the presence of synchronous causal cycles, if there are any.

## Conclusion

To our knowledge, this is the first study to model direct causal pathways between socio-affective abilities, motivation, and social and occupational functioning in an early schizophrenia sample, using a data-driven causal discovery analysis. These findings have high clinical relevance and underscore the importance of specifically and vigorously targeting social cognitive processes and motivation as early as possible in the course of treatment to enhance social and occupational recovery. In addition, our findings suggest that promoting social functioning may have continued downstream effects on motivation, socio-affective capacities, and occupational functioning, and should also be a specific target of early intervention services.

## Supplementary material

For supplementary material accompanying this paper visit https://doi.org/10.1017/S0033291721003780. click here to view supplementary material
