# Supplementary material for: Causal pathways to social and occupational functioning in the first episode of schizophrenia: uncovering unmet treatment needs
Source: Psychol Med. 2021 Oct 8;53(5):2041–9. doi: 10.1017/S0033291721003780 (PMC10106305; doi:10.1017/S0033291721003780)
Supplement: Supplementary file 1 [file S0033291721003780sup001.docx]

**Causal Pathways to Social and Occupational Functioning in the First Episode of Schizophrenia: Uncovering Unmet Treatment Needs**

**Supplementary Methods and Results**

**1. Additional description of included variables**

The following secondary variables were included in our analysis for Study 1:

*Psychopathology:.*  The Positive and Negative Symptom Scale (PANSS) traditional three syndrome model scale scores were used for positive, negative, and general symptoms variables (Kay, Fiszbein, & Opler, 1987). Depression was measured with the Calgary Depression Scale for Schizophrenia (Addington, Addington, & Maticka-Tyndale, 1993). Overall illness severity was measured with the Clinical Global Impressions-Severity scale (Guy, 1976).

*Substance use:* Alcohol and cannabis use was measured by self-report of the number of days in the preceding month that each substance was used.

*Psychological well-being:* Three self-report measures of psychological well-being were used in Study 1. The well-being variable represents the total score from an 18-item subset of the modified version of the Scales of Psychological Well-being developed by Carol Ryff. The 18-item version was used in the RAISE-ETP study and measures six areas of self-perceived psychological well-being including environmental mastery, autonomy, personal growth, positive relationships, purpose in life, and self-acceptance (Browne et al., 2016; Ryff, 1989). The mental health recovery measure used in RAISE-ETP and our analysis is a modified version of the Young, Ensing & Bullock’s Mental Health Recovery Measure (MHRM). The RAISE-ETP modified scale uses 15 of the original 30 items, and converted from a 5 item Likert scale to a 7 item Likert scale (Browne et al., 2016; Ralph, Kidder, & Phillips, 2000; Young, Ensing, & Bullock, 1999). Perceived stigma was measured in RAISE-ETP and our analysis from a modified version of the Stigma Scale (King et al., 2007). Seven of the original 28 items were used in the modified scale which represent perceived prejudice and discrimination related to mental illness (Mueser et al., 2019).

*Beliefs about medications:* The total score for the Brief Evaluation of Medication Influences and Beliefs (BEMIB) scale was used as a measure of participant attitudes regarding adhering to antipsychotic medications. This measure has been found to correlate with objective medication adherence assessments (Dolder et al., 2004). Four items from the eight item BEMIB scale were used in RAISE-ETP and in our analysis.

**2. Causal discovery and Greedy Fast Causal Inference**

Like all statistical methods that aim to make deeper inferences from data, rather than simply describing data, causal discovery analysis makes some assumptions. The primary and most general assumption is that the observed data come from a causal process that can be accurately described with a causal model. More precisely, we assume that the data is generated by one of the models among a set of explicitly defined possible models. In this way, causal discovery analysis is similar to structural equation modeling (SEM), with the main difference being that while the SEM approach asks an expert to stipulate a small number of candidate model structures at this step, causal discovery modeling considers all possible model structures that meet some very broad criteria, e.g. all structures that contain no feedback loops, or all structures that have no unmeasured common causes, or all structures that meet both criteria. This is the primary, and perhaps only, distinction between SEM analysis and causal discovery analysis, and so causal discovery does not stand on ground any less firm than SEMs do generally.

It must also be emphasized that there is a large difference between the SEM approach and causal discovery, stemming from the fact that the space of all models meeting causal discovery’s weak restrictions is extremely large. In fact, the space of models that causal discovery considers grows at a super exponential rate with the number of variables in the data set. If x is the number of variables, then the number of models typically considered by causal discovery grows at a rate proportional to 2^(x^2). As an example, with only 5 variables there are 29,281 possible directed acyclic graphs (DAGs), graphical structures which contain directed edges connecting nodes that represent observed variables, with no feedback loops formed by the edges. These DAGs correspond to the different structures that many causal discovery algorithms consider might have generated the data. With 10 variables, the number of DAGs has already grown to 4,175,098,976,430,598,143. For many data sets, the number of possible DAGs is too many to fit a SEM to each one of them and compare fit statistics.

Causal discovery analysis attempts to resolve this problem by using advanced computing strategies to compute these statistics more efficiently, and/or by rapidly removing large numbers of models from consideration without testing them individually. Greedy Fast Causal Inference (GFCI), the causal discovery analysis method used in this paper, uses the latter approach. It first uses an intelligent search procedure to selectively fit models as SEMs, iteratively modifying each SEM to optimize model fit statistics, until it cannot improve the fit statistics any further. Our implementation of GFCI for this project used the Bayesian Information Criterion (BIC) fit statistic for linear Gaussian models. The BIC score is a penalized likelihood score defined by:

BIC = - 2 ln (L) + k ln(n)

k is the number of free parameters in the model, for linear Gaussian SEMs this is the number of variables plus the number of edges. n is the sample size. The likelihood, L, is the probability of the data given the model, after fitting the model’s free parameters to the data. Lower BIC scores are preferred to higher BIC scores, forcing a trade-off between the model’s ability to plausibly generate the observed data and the model’s complexity.

For each model structure tested, the algorithm fits an appropriate multivariate Gaussian distribution to the data. Since this is a distribution over different possible values the data could take, it calculates a specific probability value for our actual data set (the likelihood). The BIC transforms the likelihood by taking its natural log, and then adds a penalty term to prevent overfitting with unnecessarily complex models. This process is repeated while the algorithm systematically searches through the space of possible models by adding and removing individual edges, until it identifies a model structure that appears to have the best BIC.

After identifying a model structure that optimizes the BIC, GFCI then goes a step further by considering that there may be unmeasured common causes. Unmeasured common causes can be explicitly modeled with individual SEMs, but again the space of models being considered is too large for this. At present, the primary approach to unmeasured common causes among the many causal discovery analysis methods is to use hypothesis testing rather than model fitting. Underlying theoretical work has been done identifying an exhaustive collection of statistical signals that indicate the presence or absence of unmeasured common causes.

For example, consider the models in the figure below. These models all have 4 measured hypothetical variables, A, B, C, and D, and model 4 also has one unmeasured variable, L. There is always an edge from A to B, and from D to C, but the relationship between B and C is different in each model.

A

B

C

D

A

B

C

D

A

B

C

D

A

B

C

D

1

2

3

4

Let’s consider how a causal discovery analysis algorithm would use tests to determine what is the most plausible causal model. If the algorithm sees in the data that B and C are significantly correlated, then it can drop model 3. Since in model 3 there is no causal pathway that connects B and C, that model would not be able to explain why B and C are correlated. Models 1, 2, and 4, can explain this correlation, so they are retained for consideration. If the algorithm then finds that B and D are significantly correlated conditional on C, or in other words that the partial correlation of B and D given C is significant, then it can drop model 2. In model 2, conditioning on C will separate B from D, implying that B and D should be uncorrelated given C, but that contradicts what we found in the data. That leaves the algorithm with models 1 and 4 still under consideration. It can then test whether A and C are correlated after conditioning on B. If it finds that A and C are correlated after conditioning on B, then it drops model 1 from consideration, as B blocks the path from A to C in model 1. This leaves only model 4, where B and C both have two arrowheads pointing at them, which can explain the above-mentioned conditional dependencies found in the hypothetical data.

In fact, this combination of testable statistical features descriptively found in the data cannot be explained by any DAG that does not have an unmeasured common cause of B and C. This sort of logical inference has been encoded into a collection of structural inference rules that can be iteratively applied, guiding a sequence of hypothesis tests that continuously refines the model by considering possible ways that unmeasured variables, like L in the above example, might influence the descriptive statistics found in the data. For a detailed list of these rules see citations (Spirtes, Glymour, & Scheines, 2000; Zhang, 2008). GFCI applies these rules in its second step, refining the initial graph learned in its first step by making adjustments based on hypothesis tests checking for specific interactions from unmeasured common causes, like that in the example given previously. The final product is a graph representing a collection of DAGs, which may include unmeasured variables in them. It is a collection, rather than a single DAG, because even with all of the above, some models can still be indistinguishable from each other when using linear Gaussian model fit and conditional independence tests on the observed variables. This collection is then graphically represented as a Partial Ancestral Graph (PAG), which has a variety of different edges that capture the potentially multiple possible relationships that two connected variables have across the different DAGs in the collection. For example, if the DAGs in the collection include a graph where A causes B, a graph where A doesn’t cause B but there is an unmeasured common cause of A and B, and a graph where A causes B and there is also an unmeasured common cause of A and B, then the PAG would use a specific edge to represent this collection of possible relationships between A and B which is typically visualized as A o--> B (see Figure 1 in the main text for a description of edge types and their interpretations).

**3. SEM comparison and sensitivity analyses (Study 1)**

**SEM comparison**

A previous factor analysis found that the items on the Quality of Life scale that comprise the motivation and socio-affective capacity variables used in this study load together during factor analysis (Mueser et al., 2017). Factor analysis does not necessarily indicate that motivation and socio-affective capacity are the same entity and separating them may have clinical relevance.

If there is an unmeasured common cause between motivation and socio-affective capacity due to redundancy of measurement or overlapping traits, GFCI should identify this as the best fit in the model search. To ensure that our results are not due to overlapping traits represented by the motivation and socio-affective capacity variables we directly tested the separateness of these variables (i.e., that the observed relationship between motivation and socio-affective capacity in our graph was not better explained by a shared latent trait) by comparing model fit statistics from our original SEM to a SEM in which the relationship between motivation and socio-affective capacity at baseline and six-months is modeled as a latent variable. Model comparisons were performed using hypothesis tests for non-nested models described in Vuong using the “nonnest2” package in R (Vuong, 1989).

Results of this analysis indicated that our original model had significantly better fit than the alternative model (z = 5.97, p = 1.2e-0.9). The Comparative Fit Index (CFI) and Root Mean Square Error (RMSEA) of both models are provided below.

|  | Original model | Alternative model |
| --- | --- | --- |
| CFI | 0.884 | 0.848 |
| RMSEA | 0.066 | 0.076 |

**Sensitivity analysis**

We completed two sensitivity analyses to ensure our results are not driven by measurement overlap. First, we excluded the socio-affective capacity variable from the model to assess whether the remaining structure of the functional outcomes subgraph was dependent on socio-affective capacity. We found that dropping socio-affective capacity did not impact the relationships in the graph, except for a change in the edge orientation between baseline motivation and Clinical Global Impressions (Figure S1). This edge did not survive the bootstrap and jackknife assessment for graph stability, indicating that it is a weak and unstable relationship in the alternative graph.

Second, we dropped PANSS item N3 measuring engagement with the interviewer from the PANSS negative symptom composite score due to measurement overlap with the socio-affective capacity variable. The original relationships were largely maintained in the resulting graph (Figure S2). In the alternative graph there is an added edge indicating that baseline negative symptoms are a direct or indirect cause of baseline social functioning. This edge did not survive the bootstrap and jackknife assessment for graph stability, indicating that it is a weak and unstable relationship.

| **4. Supplemental Tables**  Table S1. Demographic and variable descriptive statistics for included and excluded RAISE-ETP participants | | | | |
| --- | --- | --- | --- | --- |
|  | **Included** (N=276) | | **Excluded** (N=128) | |
|  | Mean (SD) or % | | Mean (SD) or % | |
| **Demographics** |  |  |  |  |
| Age (years) | 23 | (4.9) | 23 | (5.4) |
| Gender: male | 74.3 |  | 68.8 |  |
| Race |  |  |  |  |
| American Indian or Alaskan Native | 5.8 |  | 3.9 |  |
| Asian | 2.9 |  | 3.1 |  |
| Black | 35.1 |  | 43.0 |  |
| Native Hawaiian or Pacific Islander | 0.4 |  | 0.0 |  |
| White | 55.8 |  | 50.0 |  |
| Ethnicity: Hispanic or Latino | 18.5 |  | 17.2 |  |
| **Baseline Variables** |  |  |  |  |
| Treatment Assignment to Coordinated Specialty Care | 43.1 |  | 48.4 |  |
| QLS social functioning | 19.9 | (8.6) | 19.5 | (8.9) |
| QLS occupational functioning | 5.7 | (6.7) | 5.3 | (6.1) |
| QLS motivation | 7.7 | (3.6) | 7.4 | (3.5) |
| QLS socio-affective capacity | 8.0 | (2.2) | 7.6 | (2.1) |
| Neurocognitive Composite Z score | 0.04 | (1.0) | 0.0 | (1.0) |
| PANSS positive symptoms | 18.9 | (5.2) | 18.4 | (5.2) |
| PANSS negative symptoms | 20.1 | (5.2) | 20.4 | (5.5) |
| PANSS general symptoms | 37.6 | (7.7) | 37.8 | (8.8) |
| Calgary Depression Scale for Schizophrenia | 4.4 | (3.9) | 5.3 | (4.8) |
| Clinical global impressions of severity | 4.1 | (0.8) | 4.0 | (0.8) |
| Duration of untreated psychosis (days) | 195.7 | (271.9) | 187.3 | (240.4) |
| Days of alcohol use in last month | 1.9 | (4.7) | 1.8 | (4.1) |
| Days of cannabis use in last month | 3.2 | (7.6) | 2.7 | (7.0) |
| Well-being scale total score | 4.0 | (0.9) | 3.9 | (1.0) |
| Mental health recovery measure total score | 73.3 | (18.1) | 73.4 | (19.6) |
| Stigma scale total score | 4.0 | (1.2) | 3.9 | (1.3) |
| Brief evaluation of medication influences  and beliefs total score* | 4.9 | (1.0) | 5.1 | (1.0) |
| * p=0.03, t=-2.2  PANSS = Positive and Negative Symptom Scale  ^ζ^Neurocognitive composite from the Brief Assessment of Cognition  QLS = Quality of Life Scale; PANSS = Positive and Negative Symptom Scale | | | | |

Table S2. Study 1 RAISE-ETP sample causal effect sizes

| **Edge Type in PAG** | **Nodes** | | **Raw Effect Size**  **(standard error)** | | **95% Confidence interval** | **Standardized effect size (standard error)** | | **95% Confidence interval** | **Z-score** | **p-Value** |
| --- | --- | --- | --- | --- | --- | --- | --- | --- | --- | --- |
|  | Node 1 | Node 2 |  |  |  |  |  |  |  |  |
| O🡪 | Age (years) | Days alcohol use in last month | 0.07 | (0.02) | 0.03, 0.12 | 0.18 | (0.06) | 0.07, 0.29 | 3.1 | 0.002 |
|  | Age (years) | DUP | 0.02 | (0.004) | 0.01, 0.03 | 0.28 | (0.06) | 0.16, 0.39 | 4.5 | 0 |
| 🡪 | CGI | PANSS Positive | 4.57 | (0.28) | 4.03, 5.11 | 0.71 | (0.04) | 0.63, 0.80 | 16.6 | 0 |
|  | PANSS Negative | Socio-affective Capacity 6 Month | -0.17 | (0.02) | -0.21, -0.13 | -0.38 | (0.05) | -0.47, -0.3 | -8.1 | 0 |
| 🡪 | CGI | Motivation | -1.41 | (0.21) | -1.82, -1.0 | -0.32 | (0.05) | -0.42, -0.23 | -6.74 | 0 |
|  | Days alcohol use in last month | Days cannabis use in last month | 0.34 | (0.09) | 0.16, 0.53 | 0.21 | (0.06) | 0.10, 0.33 | 3.6 | 0 |
|  | Depression | PANSS General | 0.89 | (0.10) | 0.69, 1.1 | 0.46 | (0.05) | 0.36, 0.57 | 8.7 | 0 |
|  | Depression | Well-being scale total score | -0.08 | (0.01) | -0.10, -0.06 | -0.37 | (0.06) | -0.48, -0.26 | -6.7 | 0 |
|  | DUP | Depression | 1.2 | (0.27) | 0.70, 1.76 | 0.26 | (0.06) | 0.15, 0.38 | 4.5 | 0 |
|  | Global Cognition | PANSS Positive | 1.09 | (0.24) | 0.63, 1.55 | 0.2 | (0.04) | 0.12, 0.28 | 4.65 | 0 |
|  | Mental Health Recovery Measure total score | Medication Beliefs | 0.01 | (.003) | .007, .002 | 0.25 | (0.06) | 0.13, 0.36 | 4.27 | 0 |
|  | Motivation | Global Cognition | 0.08 | (0.02) | 0.05, 0.12 | 0.32 | (0.06) | 0.20, 0.43 | 5.5 | 0 |
|  | Motivation | Occupational Functioning | 0.96 | (0.10) | 0.77, 1.15 | 0.51 | (0.05) | 0.41, 0.61 | 9.7 | 0 |
|  | Motivation | Social Functioning | 1.45 | (0.12) | 1.22, 1.68 | 0.6 | (0.05) | 0.50, 0.70 | 12.3 | 0 |
|  | Motivation 6 Month | Socio-affective Capacity 6 Month | 0.24 | (0.03) | 0.18, 0.3 | 0.37 | (0.05) | 0.28, 0.46 | 7.8 | 0 |
|  | Motivation 6 Month | Occupational Functioning 6 Month | 0.92 | (0.10) | 0.73, 1.12 | 0.45 | (0.05) | 0.35, 0.54 | 9.3 | 0 |
|  | Occupational Functioning | Occupational Functioning 6 Month | 0.36 | (0.05) | 0.26, 0.47 | 0.33 | (0.05) | 0.23, 0.42 | 6.8 | 0 |
|  | PANSS General | PANSS Negative | 0.35 | (0.04) | 0.28, 0.42 | 0.51 | (0.05) | 0.41, 0.61 | 9.9 | 0 |
|  | PANSS General | CGI | 0.07 | (.007) | 0.06. 0.08 | 0.68 | (0.04) | 0.59, 0.76 | 15.3 | 0 |
|  | PANSS Negative | Socio-affective Capacity | -0.27 | (0.02) | -0.31, -0.23 | -0.65 | (0.05) | -0.74, -0.56 | -14.2 | 0 |
|  | Social Functioning | Social Functioning 6 Month | 0.60 | (0.06) | 0.49, 0.71 | 0.53 | (0.05) | 0.43, 0.63 | 10.3 | 0 |
|  | Social Functioning 6 Month | Motivation 6 Month | 0.22 | (0.02) | 0.18, 0.25 | 0.6 | (0.05) | 0.49, 0.68 | 12.1 | 0 |
|  | Socio-affective Capacity | Motivation | 0.76 | (0.08) | 0.60, 0.91 | 0.46 | (0.05) | 0.37, 0.55 | 9.65 | 0 |
|  | Well-being Scale total score | Mental Health Recovery Measure total score | 16.07 | (0.83) | 14.45, 17.7 | 0.76 | (0.04) | 0.68, 0.84 | 19.4 | 0 |
|  | Well-being Scale total score | Stigma scale total score | -0.52 | (0.08) | -0.67, -0.38 | -0.38 | (0.06) | -0.49, -0.27 | -6.9 | 0 |
| Age is log transformed. CGI = Clinical Global Impressions of Severity; DUP = duration of untreated psychosis, measured in days (log transformed); Depression = Calgary Depression Scale for Schizophrenia; Medication Beliefs = Brief Evaluation of Medication Influences and Beliefs total score; PANSS General = Positive and Negative Symptom Scale General Symptoms total score; PANSS Negative = Positive and Negative Symptom Scale Negative Symptoms total score; PANSS Positive = Positive and Negative Symptom Scale Positive Symptoms total score | | | | | | | | | | |

| Table S3. Re-sampling analyses for Study 1 (RAISE-ETP) and Study 2 (CATIE) samples | | | | | | | | | |
| --- | --- | --- | --- | --- | --- | --- | --- | --- | --- |
| **Jack-knife re-sampling analysis for Study 1 RAISE-ETP sample** | | | | | | | | | |
| **Edge Type in PAG** | **Nodes** | | **Proportion of 1,000 jack-knife resamples with edge type** | | | | | | |
| o🡪 | Node 1 | Node 2 | 🡪 | 🡨 | o🡪 | 🡨o | o--o | 🡨🡪 | No edge |
|  | Age | Days Alcohol Use | 0 | 0 | **0.666** | 0 | 0 | 0 | 0.334 |
|  | Age | DUP | 0 | 0 | **0.999** | 0 | 0 | 0 | 0.001 |
| 🡪 | CGI | Motivation | **0.949** | 0.019 | 0.004 | 0.001 | 0 | 0 | .027 |
|  | CGI | PANSS Positive | **0.837** | 0.094 | 0.002 | 0.047 | 0.012 | .008 | 0 |
|  | Days Alcohol Use | Days Cannabis Use | **0.388** | 0 | 0 | 0 | 0.256 | 0 | 0.356 |
|  | Depression | PANSS General | **0.942** | 0.055 | 0 | 0 | 0.003 | 0 | 0 |
|  | Depression | Well-being Total Score | **0.931** | 0.06 | 0 | 0 | 0.003 | 0 | 0.006 |
|  | DUP | Depression | **0.886** | 0.012 | 0 | 0 | 0 | 0 | 0.102 |
|  | Global Cognition | PANNS Positive | **0.668** | 0 | 0.005 | 0 | 0 | 0 | 0.327 |
|  | Mental Health Recovery Measure total score | Medication Beliefs | **0.568** | 0 | 0 | 0 | 0 | 0 | 0.432 |
|  | Motivation | Global Cognition | **0.973** | 0 | 0 | 0 | 0.005 | 0 | 0.022 |
|  | Motivation | Occupational Functioning | **0.995** | 0 | 0 | 0 | 0.005 | 0 | 0 |
|  | Motivation | Social Functioning | **0.995** | 0 | 0 | 0 | 0.005 | 0 | 0 |
|  | Motivation 6 Month | Socio-affective capacity 6 Month | **0.836** | 0.163 | 0 | 0 | 0 | 0 | 0.001 |
|  | Motivation 6 Month | Occupational Functioning 6 Month | **1** | 0 | 0 | 0 | 0 | 0 | 0 |
|  | Occupational Functioning | Occupational Functioning 6 Month | **0.995** | 0 | 0.005 | 0 | 0 | 0 | 0 |
|  | PANSS General | PANSS Negative | **0.799** | 0.002 | 0 | 0.09 | 0 | 0.006 | 0.103 |
|  | PANSS General | CGI | **0.87** | 0.045 | 0.001 | 0 | 0.004 | 0 | 0.08 |
|  | PANSS Negative | Socio-affective capacity | **0.862** | 0.002 | 0.001 | 0.001 | 0.134 | 0 | 0 |
|  | PANSS Negative | Socio-affective capacity 6 Month | **0.864** | 0 | 0.135 | 0 | 0 | 0 | 0.001 |
|  | Social Functioning | Social Functioning 6 Months | **0.995** | 0 | 0.005 | 0 | 0 | 0 | 0 |
|  | Social Functioning 6 Month | Motivation 6 Month | **0.875** | 0.125 | 0 | 0 | 0 | 0 | 0 |
|  | Socio-affective capacity | Motivation | **0.85** | 0.014 | 0.131 | 0.001 | 0.004 | 0 | 0 |
|  | Well-being Scale total score | Mental Health Recovery Measure total score | **0.991** | 0.006 | 0 | 0 | 0.003 | 0 | 0 |
|  | Well-being Scale total score | Stigma Scale total score | **0.997** | 0 | 0 | 0 | 0.003 | 0 | 0 |
| **Bootstrap re-sampling analysis for Study 1 RAISE-ETP sample** | | | | | | | | | |

| **Edge Type in PAG** | **Nodes** | | **Proportion of 1,000 bootstrap resamples with edge type** | | | | | | |
| --- | --- | --- | --- | --- | --- | --- | --- | --- | --- |
| o🡪 | Node 1 | Node 2 | 🡪 | 🡨 | o🡪 | 🡨o | o--o | 🡨🡪 | No edge |
|  | Age | Days Alcohol Use | 0 | 0 | **0.553** | 0 | 0 | 0 | 0.467 |
|  | Age | DUP | 0 | 0 | **0.857** | 0 | 0 | 0 | 0.143 |
| 🡪 | CGI | Motivation | **0.412** | 0.054 | 0.045 | 0.003 | 0.009 | 0.002 | 0.475 |
|  | CGI | PANSS Positive | **0.443** | 0.204 | 0.089 | 0.111 | 0.052 | 0.101 | 0 |
|  | Days Alcohol Use | Days Cannabis Use | 0.277 | 0.031 | 0 | 0.014 | 0.222 | 0 | **0.456** |
|  | Depression | PANSS General | **0.491** | 0.442 | 0.022 | 0.012 | 0.033 | 0 | 0 |
|  | Depression | Well-being Total Score | **0.596** | 0.169 | 0.01 | 0.004 | 0.033 | 0 | 0.188 |
|  | DUP | Depression | 0.287 | 0.141 | 0 | 0.009 | 0.005 | 0 | **0.558** |
|  | Global Cognition | PANNS Positive | 0.301 | 0.015 | 0.072 | 0.003 | 0 | 0 | **0.609** |
|  | Mental Health Recovery Measure total score | Medication Beliefs | 0.396 | 0.004 | 0.001 | 0 | 0.016 | 0 | **0.583** |
|  | Motivation | Global Cognition | **0.55** | 0.002 | 0.003 | 0.054 | 0.08 | 0.001 | 0.31 |
|  | Motivation | Occupational Functioning | **0.807** | 0.031 | 0.013 | 0.041 | 0.107 | 0.001 | 0 |
|  | Motivation | Social Functioning | **0.762** | 0.104 | 0.027 | 0.008 | 0.098 | 0 | 0.001 |
|  | Motivation 6 Month | Socio-affective capacity 6 Month | **0.5** | 0.367 | 0 | 0 | 0 | 0 | 0.133 |
|  | Motivation 6 Month | Occupational Functioning 6 Month | **0.973** | 0.023 | 0 | 0 | 0 | 0 | 0.004 |
|  | Occupational Functioning | Occupational Functioning 6 Month | **0.838** | 0 | 0.147 | 0 | 0 | 0 | 0.015 |
|  | PANSS General | CGI | **0.52** | 0.219 | 0.01 | 0.012 | 0.047 | 0.002 | 0.19 |
|  | PANSS General | PANSS Negative | **0.333** | 0.176 | 0.002 | 0.176 | 0.026 | 0.019 | 0.268 |
|  | PANSS Negative | Socio-affective capacity | **0.426** | 0.246 | 0.036 | 0.027 | 0.264 | 0.001 | 0 |
|  | PANSS Negative | Socio-affective capacity 6 Month | **0.65** | 0 | 0.288 | 0 | 0 | 0.002 | 0.06 |
|  | Social Functioning | Social Functioning 6 Month | **0.889** | 0 | 0.108 | 0 | 0 | 0 | 0.003 |
|  | Social Functioning 6 Month | Motivation 6 Month | **0.726** | 0.273 | 0 | 0 | 0 | 0.001 | 0 |
|  | Socio-affective capacity | Motivation | **0.368** | 0.239 | 0.196 | 0.03 | 0.082 | 0.016 | 0.069 |
|  | Well-being Scale total score | Mental Health Recovery | **0.791** | 0.157 | 0.001 | 0.003 | 0.048 | 0 | 0 |
|  | Well-being Scale total score | Stigma Scale total score | **0.818** | 0.065 | 0.008 | 0.002 | 0.039 | 0 | 0.068 |
| **Jack-knife re-sampling analysis for Study 2 CATIE sample** | | | | | | | | | |
| **Edge Type in PAG** | **Nodes** | | **Proportion of 1,000 jack-knife resamples with edge type** | | | | | | |
| o--o | Node 1 | Node 2 | 🡪 | 🡨 | o🡪 | 🡨o | o--o | 🡨🡪 | No edge |
|  | Depression | PANSS General | 0.002 | 0.042 | 0.052 | 0 | **0.904** | 0 | 0 |
|  | Motivation | Occupational Functioning | 0.062 | 0 | 0 | 0 | **0.938** | 0 | 0 |
|  | Motivation | Social Functioning | 0.028 | 0.034 | 0.218 | 0 | **0.72** | 0 | 0 |
|  | PANSS General | PANSS Negative | 0 | 0.034 | 0 | 0.062 | **0.904** | 0 | 0 |
|  | PANSS General | PANSS Positive | 0.086 | 0 | 0 | 0.01 | **0.904** | 0 | 0 |
|  | PANSS Negative | Socio-affective capacity | 0 | 0.034 | 0 | 0 | **0.955** | 0 | 0.011 |
|  | PANSS Negative | Global Cognition | 0.034 | 0 | 0 | 0 | **0.94** | 0 | 0.026 |
|  | Socio-affective capacity | Motivation | 0 | 0.044 | 0.018 | 0 | **0.938** | 0 | 0 |
| o🡪 | Global Cognition | CGI | 0.02 | 0.022 | **0.599** | 0 | 0 | 0 | 0.359 |
|  | Motivation | Motivation 6 Months | 0.038 | 0 | **0.593** | 0 | 0 | 0 | 0.369 |
|  | PANSS Positive | CGI | 0.084 | 0 | **0.903** | 0 | 0.007 | 0 | 0.006 |
|  | Occupational Functioning | Occupational Functioning 6 Month | 0.062 | 0 | **0.938** | 0 | 0 | 0 | 0 |
|  | Social Functioning | Social Functioning 6 Month | 0.28 | 0 | **0.72** | 0 | 0 | 0 | 0 |
|  | Socio-affective capacity | Socio-affective capacity 6 Month | 0.038 | 0 | **0.789** | 0 | 0 | 0 | 0.173 |
| 🡪 | CGI | Facial Affect Recognition | **0.641** | 0 | 0 | 0.005 | 0.008 | 0 | 0.346 |
|  | Motivation 6 Month | Socio-affective capacity 6 Month | **0.809** | 0.163 | 0 | 0 | 0.002 | 0 | 0.026 |
|  | Motivation 6 Month | Occupational Functioning 6 Month | **0.897** | 0.103 | 0 | 0 | 0 | 0 | 0 |
|  | Social Functioning 6 Month | Motivation 6 Month | **0.843** | 0.012 | 0 | 0 | 0 | 0 | 0.145 |
| No edge | Age (years) | Age at first antipsychotic use | 0 | 0.001 | 0.171 | 0.011 | **0.817** | 0 | 0 |
| **Bootstrap re-sampling analysis for Study 2 CATIE sample** | | | | | | | | | |
| **Edge Type in PAG** | **Nodes** | | **Proportion of 1,000 bootstrap resamples with edge type** | | | | | | |
| o--o | Node 1 | Node 2 | 🡪 | 🡨 | o🡪 | 🡨o | o--o | 🡨🡪 | No edge |
|  | Depression | PANSS General | 0.11 | **0.342** | 0.182 | 0.009 | 0.27 | 0 | 0.087 |
|  | Motivation | Occupational Functioning | 0.419 | 0.008 | 0.025 | 0.014 | **0.501** | 0 | 0.033 |
|  | Motivation | Social Functioning | 0.212 | 0.238 | 0.151 | 0.007 | **0.392** | 0 | 0 |
|  | PANSS General | PANSS Negative | 0.066 | **0.333** | 0.028 | 0.271 | 0.296 | 0.006 | 0 |
|  | PANSS General | PANSS Positive | **0.384** | 0.069 | 0.003 | 0.218 | 0.32 | 0.006 | 0 |
|  | PANSS Negative | Socio-affective capacity | 0.054 | 0.186 | 0.034 | 0.113 | **0.396** | 0 | 0.217 |
|  | PANSS Negative | Global Cognition | 0.267 | 0.03 | 0.017 | 0.012 | **0.389** | 0 | 0.285 |
|  | Socio-affective capacity | Motivation | 0.061 | 0.31 | 0.077 | 0.02 | **0.521** | 0 | 0.011 |
| o🡪 | Global Cognition | CGI | 0.074 | 0.092 | 0.16 | 0.002 | 0.03 | 0 | **0.642** |
|  | Motivation | Motivation 6 Months | 0.229 | 0 | 0.272 | 0 | 0 | 0.001 | **0.498** |
|  | PANSS Positive | CGI | **0.324** | 0.067 | **0.324** | 0.011 | 0.12 | 0 | 0.154 |
|  | Occupational Functioning | Occupational Functioning 6 Month | 0.456 | 0 | **0.492** | 0 | 0 | 0 | 0.052 |
|  | Social Functioning | Social Functioning 6 Month | **0.599** | 0 | 0.398 | 0 | 0 | 0.003 | 0 |
|  | Socio-affective capacity | Socio-affective capacity 6 Month | 0.229 | 0 | 0.347 | 0 | 0 | 0 | **0.424** |
| 🡪 | CGI | Facial Affect Recognition | 0.268 | 0.02 | 0.003 | 0.031 | 0.063 | 0 | **0.615** |
|  | Motivation 6 Month | Socio-affective capacity 6 Month | **0.546** | 0.206 | 0 | 0 | 0.005 | 0 | 0.243 |
|  | Motivation 6 Month | Occupational Functioning 6 Month | **0.636** | 0.34 | 0.001 | 0 | 0.001 | 0 | 0.022 |
|  | Social Functioning 6 Month | Motivation 6 Month | **0.509** | 0.131 | 0 | 0 | 0 | 0.001 | 0.359 |
| No edge | Age (years) | Age at first antipsychotic use | 0 | 0.138 | 0.31 | 0.11 | **0.43** | 0.012 | 0 |
| Age is log transformed in RAISE sample. CGI = Clinical Global Impressions of Severity; DUP = duration of untreated psychosis, measured in days (log transformed); Depression = Calgary Depression Scale for Schizophrenia; Medication Beliefs = Brief Evaluation of Medication Influences and Beliefs total score; PANSS General = Positive and Negative Symptom Scale General Symptoms total score; PANSS Negative = Positive and Negative Symptom Scale Negative Symptoms total score; PANSS Positive = Positive and Negative Symptom Scale Positive Symptoms total score | | | | | | | | | |

**References**

Addington, D., Addington, J., & Maticka-Tyndale, E. (1993). Assessing depression in schizophrenia: the Calgary Depression Scale. *Br J Psychiatry Suppl.*, *22*, 39–44.

Browne, J., Penn, D. L., Meyer-Kalos, P. S., Mueser, K. T., Estroff, S. E., Brunette, M. F., … Kane, J. M. (2016). Psychological well-being and mental health recovery in the NIMH RAISE early treatment program. *Schizophrenia Research*, *185*, 167–162. https://doi.org/10.1016/j.schres.2016.11.032

Dolder, C. R., Lacro, J. P., Warren, K. A., Golshan, S., Perkins, D. O., & Jeste, D. V. (2004). Brief evaluation of medication influences and beliefs: development and testing of a brief scale for medication adherence. *Journal of Clinical Psychopharmacology*, *24*(4), 404–409. https://doi.org/10.1097/01.jcp.0000130554.63254.3a

Guy, W. (1976). *ECDEU assessment manual for psychopharmacology.* US Department of Health, Education, and Welfare, Public Health Service, Alcohol, Drug Abuse, and Mental Health Administration, National Institute of Mental Health, Psychopharmacology Research Branch, Division of Extramural Research Programs.

Kay, S., Fiszbein, A., & Opler, L. (1987). The Positive and Negative Syndrome Scale (PANSS) for Schizophrenia. *Schizophrenia Bulletin*, *13*, 261–276.

King, M., Dinos, S., Shaw, J., Watson, R., Stevens, S., Passetti, F., … Serfaty, M. (2007). The Stigma Scale: development of a standardised measure of the stigma of mental illness. *The British Journal of Psychiatry : The Journal of Mental Science*, *190*, 248–254. https://doi.org/10.1192/bjp.bp.106.024638

Mueser, K. T, Kim, M., Addingon, J., Mcgurk, S., Pratt, S., & Addington, D. (2017). Confirmatory factor analysis of the quality of life scale and new proposed factor structure for the quality of life scale-revised. *Schizophrenia Research*, *181*, 117–123. https://doi.org/10.1016/j.schres.2016.10.018

Mueser, K. T., DeTore, N. R., Kredlow, M. A., Bourgeois, M. L., Penn, D. L., & Hintz, K. (2019). Clinical and demographic correlates of stigma in first-episode psychosis: the impact of duration of untreated psychosis. *Acta Psychiatrica Scandinavica*, (1), 1–10. https://doi.org/10.1111/acps.13102

Ralph, R., Kidder, K., & Phillips, D. (2000). Can we measure recovery? A compendium of recovery and recovery-related instruments. Cambridge, MA: Human Services Research Institute. U.S. Department of Health and Human Services.

Ryff, C. D. (1989). Happiness is everything, or is it? Explorations on the meaning of psychological well-being. *Journal of Personality and Social Psychology*, *57*(6), 1069–1081. https://doi.org/10.1037/0022-3514.57.6.1069

Spirtes, P., Glymour, C. N., & Scheines, R. (2000). *Causation, prediction, and search.* MIT Press.

Vuong, Q. H. (1989). Likelihood Ratio Tests for Model Selection and Non-Nested Hypotheses. *Econometrica*, *57*(2), 307. https://doi.org/10.2307/1912557

Young, S., Ensing, D. S., & Bullock, W. A. (1999). The Mental Health Recovery Measure. Toledo, OH: University of Toledo, Department of Psychology.

Zhang, J. (2008). On the completeness of orientation rules for causal discovery in the presence of latent confounders and selection bias. *Artificial Intelligence*, *172*(16–17), 1873–1896. https://doi.org/10.1016/J.ARTINT.2008.08.001
